# Concurrent detection of bovine viral diarrhoea virus and bovine herpesvirus-1 in bulls’ semen and their effect on semen quality

**DOI:** 10.1080/23144599.2020.1850197

**Published:** 2020-12-17

**Authors:** Rania S. El-Mohamady, Tahani S. Behour, Z.M. Rawash

**Affiliations:** a Viral Diseases Research Unit, Animal Reproduction Research Institute (ARRI), Agricultural Research Center (ARC), Giza, Egypt; b Biotechnology Research Unit, Animal Reproduction Research Institute (ARRI), Agricultural Research Center (ARC), Giza, Egypt; c Artificial Insemination and Embryo Transfer Department, Animal Reproduction Research Institute (ARRI), Agricultural Research Center (ARC), Giza, Egypt

**Keywords:** BVDV, BoHV-1, semen, isolation, FAT, real-time PCR

## Abstract

Reproductive diseases may have destructive effects on the fertility of cattle. Bovine viral diarrhoea virus (BVDV) and bovine herpes virus-1 (BoHV-1) are potent viral pathogens linked to reproduction. Thus, the aim of this study was to utilize raw semen samples for conventional and molecular detection of BVDV and BoHV-1, simultaneously. Additionally, the effect of virus infection on the semen quality of naturally infected bulls has been investigated. Therefore, 40 bulls were employed for semen collection, evaluation and testing for both viruses by virus isolation, direct fluorescent antibody technique (FAT) and SYBR Green real-time PCR assay. In virus isolation results, no cytopathic effect (CPE) was observed for BVDV on cell culture whereas, eight (20%) samples displayed characteristic grape-like clusters of cells for BoHV-1. By direct FAT, 12 (30%) positive BVDV and 8 (20%) positive BoHV-1 samples were confirmed. SYBR Green real-time PCR analysis using 48 h inoculated semen samples revealed 14 (35%) and 8 (20%) positive samples for BVDV and BoHV-1, respectively. Statistical analysis of semen evaluation parameters showed a significant difference between viral-infected and free groups represented by increased sperm abnormalities and decreased sperm motility, liveability and concentration. However, there was no significant difference among BVDV, BoHV-1 and mixed-infected groups. The study concluded that BVDV and/or BoHV- 1 infected bulls expressed low semen quality. Real-time PCR was confirmed to be the ideal laboratory assay for detection of both viruses in semen.

## 1. Introduction

Reproductive diseases can cause destructive consequences on fertility in both male and female cattle. Bull fertility can be impaired temporarily or permanently, depending on the type of infectious agent and the lesions produced on reproductive organs [1]. Several viruses have been isolated from bovine semen. These viruses are freely present in semen plasma or attach to the sperm head. Thus, contaminated semen may not only infect cows but also lead to the risk of direct oocyte infection [1,2].

Bovine viral diarrhoea virus (BVDV) and bovine herpes virus-1 (BoHV-1) are significant reproduction linked to viral pathogens. They are classified as bovine aetiological agents that cause major economic losses attributed to reproductive failure, calf mortality, as well as enteric and respiratory diseases [3]. BVDV and BoHV-1 are defined as semen-transmittable viruses due to their persistence and latency, respectively [4,5].

BVDV is linked to a wide range of animal diseases. It is a member of genus *Pestivirus*, family *Flaviviridae*. Its genome is approximately 12.5 kb of single-stranded positive sense RNA [6]. It includes a particular open reading frame (ORF) flanked by a noncoding region (NCR) at the 5ʹ and 3ʹ terminals. Strains of BVDV1 and BVDV2 genotypes are more defined as cytopathic (cp) or noncytopathic (ncp) biotypes arising from the lytic action of the virus in cell culture [7]. BVDV infection is endemic in many distinct regions around the world where cattle are the natural host of the virus [8].

Reproductive tissues attacking by BVDV results in reproductive losses, ranging from a subtle reduction in reproductive efficiency to massive abortion storms in the herd. The virus preserves itself and spreads through the reproductive system in the cattle [9]. As a result, infection is connected with a reduction in the fertility of infected cattle [10]. BVDV employs bull’s seminal vesicles, prostate gland and testes for replication, resulting in a persistent testicular infection, and the virus may descend in bull’s semen following both acute and persistent infection, leading to the possible source of infection through semen [11,12]. Cows fertilized with semen from a persistently infected (PI) bull showed a lower conception rate of 38% versus 66% in those bred with semen from BVDV-free bull [13].

Bovine herpes virus-1 (BoHV-1) belongs to the family *Herpesviridae*. It is classified according to the genomic and antigenic characteristics into BoHV-1.1; BoHV-1.2 which subdivided into subtypes BoVH-1.2a and BoHV-1.2b [14]. The viral genome is double-stranded DNA, about 136,000 bp in size, and encodes for approximately 70 proteins [15]. It produces a lot of economically important reproductive problems in cattle including vulvovaginitis, endometritis and abortion in females, and balanoposthitis in males. In the bull, semen is almost contaminated by virus excretion from the infected mucosae during semen collection as the virus localized and replicates initially in the mucosae of the prepuce, penis and urethra. Following primary infection by BoHV-1, as well as infection by attenuated live vaccine strains, the virus locates the cranial or spinal ganglia of the infected animal where it persists latent for life. The viral latency condition means that clinically normal animals are lifelong carriers. Guerin et al. [16] studied the influence of BoHV-1 on some oocytes that were exposed to the virus during maturation and fertilization. Their results showed that the virus significantly reduced the in vitro fertilization (IVF) rate and raised the level of sperm decondensation abnormalities.

Testing of semen from bulls preceding admission to an artificial insemination centre became highly recommended [17]. Recent laboratory-based virus detection methods comprise virus isolation, antigen detection, identification of viral nucleic acids in addition to serological assays [18]. Unfortunately, laboratory diagnostic tests on semen have considerable restrictions as unprocessed raw semen is a tricky sample for use in the detection of viruses [19]. The virucidal and cytotoxic properties of seminal plasma on cell culture and its inhibitory actions on reverse transcriptase enzymes are crucial in isolation and detection [20]. Therefore, removal of seminal inhibitors is the key to successful utilization of semen sample. Accordingly, the aim of the current study was to utilize raw semen samples for conventional and molecular detection of BVDV and BoHV-1, simultaneously. Additionally, the effect of virus infection on the semen quality of naturally infected bulls has been investigated.

## 2. Material and methods

### 2.1. Semen collection and evaluation

Forty apparently healthy bulls, maintained on private farms, were used in the present study. The age of animals ranged from 3 to 4 years and their body weights ranged from 400 to 550 Kg. Two ejaculates were collected from each bull early in the morning using a pre-warmed artificial vagina (40°C–42°C). Immediately following collection, the ejaculates were evaluated for volume, progressive motility, concentration, percentages of dead sperms and sperms abnormalities according to Khan and Salisbury et al. [21,22]. Semen samples were collected in a screw capped plastic vials and transported on ice to the laboratory for viral investigation.

### 2.2. Virus isolation

Raw semen samples were diluted 1:10 in sterile phosphate buffer saline (PBS) before adding to cell cultures according to OIE recommendations [23]. Tested Madin-Darby bovine kidney (MDBK) cells were seeded in 24 well tissue culture (T.C) plates at a density of 10^5^ cells/well. Each well was inoculated with previously prepared sample and incubated for 2 h at the standard culture condition (37ºC, 5% CO_2_ and 85% RH). The inoculums were removed and the cells were washed with the minimum essential medium (MEM) without foetal bovine serum (FBS) prior to the addition of an appropriate volume of maintenance MEM that supplemented with 2% FBS. The plates were incubated under standard culture conditions with daily observation for cytopathogenic effects up to 5–7 days. The cells were frozen and thawed for several times and re-inoculated. Three blind passages were applied to all samples.

### 2.3. Fluorescence antibody technique (FAT)

After 48 h post-inoculation, the viral agents were identified using direct fluorescence antibody technique. Polyclonal anti-BVDV (VMRD; cat.No.CJ-F-BVD-10 µl) and polyclonal anti-BoHV-1 (VMRD; Cat. No. CJ-F- BHV-10 µl) hyperimmune serum conjugated with fluorescence isothiocyanate (FITC) were used to identify the virus agents as described by Saliki et al. [24] for BVDV and Mweene et al. [25] for BoHV-1. Inverted epi-fluorescence light and phase contrast tri-nuclear microscope with digital camera and software analysis, Nikon, Japan was used for examination.

### 2.4. Identification of viral nucleic acids

#### 2.4.1. RNA extraction and reverse transcription to cDNA for BVDV detection

Under strict laboratory precautions, total RNA was extracted from 250 μl of each 48 h cell-cultured semen sample. NADL reference strain as positive control and MDBK cell lines as negative control were also included in extraction. Extraction was achieved using QIAzol Lysis Reagent (QIAGEN) by following the manufacturer’s instruction. Extracted RNA was immediately reverse transcribed to cDNA with RevertAid First Strand cDNA Synthesis kit (Fermentas, Thermo Scientific, Germany) according to the manufacturer^,^s instruction and stored at −70°C until use.

#### 2.4.2. DNA extraction for BoHV-1 detection

Isolation of BoHV-1 DNA was done as previously described by Sambrook et al. [26]. In brief, 250 μl of each cell-cultured semen sample were added to 250 μl of Tris-EDTA buffer (10 mM Tris-HCl, 1 mM EDTA; pH 7.6) then 1% SDS and 0.3 mg/ml of proteinase K were added to the mixture and incubated at 56°C for 3 h. DNA was purified by phenol/chloroform-isoamyl alcohol and then precipitated by absolute ethanol. The DNA pellet was washed by 70% alcohol and resuspended in 20 μl nuclease-free water and stored at −70°C till used in PCR assay. During extraction, Colorado strain was included as positive control while MDBK cell lines were employed as a negative control.

#### 2.4.3. SYBR green real-time polymerase chain reaction assays

According to OIE [23] recommendations for detection of BVDV, real-time PCR amplifications were performed by using BVDV 190-F & V326 primer pair described by Hoffmann et al. [27] to amplify a 208 base-pair fragment of the 5ʹ un-translated region (5ʹ UTR) of the pestivirus genome. Each 25 μl reaction included 12.5 μl (2X) Maxima SYBR Green qPCR Master Mix (Thermo scientific, Germany), 10 pmol BVD 190-F forward primer (5ʹ-GRAGTCGTCARTGGTTCGAC-3ʹ), 10 pmol V326 reverse primer (5ʹ-TCAACTCCATGTGCCATGTAC -3ʹ), and 5 μl cDNA sample. The PCR tubes were placed in RotorGene 6000 real-time detection system (Corbett Research, Australia) programmed for the test as follows: activation step at 95°C for 10 min then 40 cycles of denaturation step at 94°C for 10 s, annealing at 58°C for 15 s and extension at 72°C for 20 s. A single fluorescence reading for each sample was taken at the extension step.

For BoHV-1, the target for PCR amplification included a 97 base-pair sequence of the glycoprotein B (gB) gene with specific forward (5ʹ-TGT-GGA-CCT-AAA-CCT-CAC-GGT-3ʹ) and reverse primers (5ʹ-GTA-GTC-GAG-CAG-ACC-CGT-GTC-3ʹ),asrecommended by OIE [28]. The amplification steps started with an activation step at 95°C for 10 min followed by 40 cycles of denaturation for 10 s at 94°C, annealing for 10 s at 60°C and extension for 15 s at 72°C. Results were expressed by calculation of the cycle threshold (Ct) which marked the cycle when the fluorescence of a given sample significantly exceeded the baseline signal. Melt curve analysis was performed at the end of each run by heating the PCR products from 60°C to 95°C. The fluorescence data were collected continuously during the melt curve program by SYBR green channel. Any sample that had a cycle threshold (Ct) value less than 40, and its specificity was confirmed by melt curve analysis, was regarded as positive.

### 2.5. Statistical analysis

Data were subjected to statistical analysis using SPSS software according to Snedecor and Cochran [29]. One-way ANOVA employing a completely randomized design.

## 3. Result

### 3.1. Virus isolation

**Figure 1.**
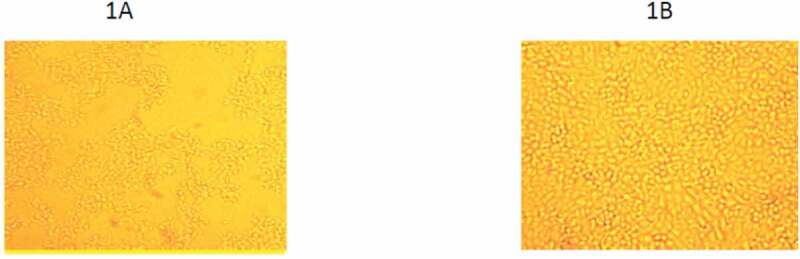
(a) Characteristic CPE of BoHV-1 on MDBK cells in the form of grape-like appearance, round and detached cells. (b) Control noninfected complete sheet of MDBK cells

After three successive passages for BoHV-1 isolation, 8(20%) out of 40 samples showed a clear cytopathic effect (CPE). It appeared as characteristic grape-like clusters of cells, which were round and aggregated together in a separated suspended manner as shown in Figure 1(a) compared with control noninfected MDBK cells (1B). After three blind passages for BVDV isolation, CPE was not observed.

### 3.2. Viral identification using direct fluorescent antibody technique

**Figure 2.**
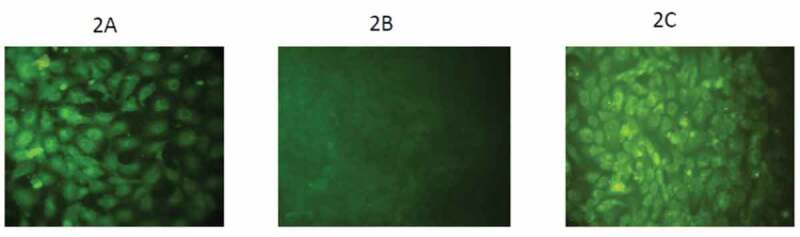
(a) MDBK cells inoculated with semen samples, treated with direct fluorescence antibody technique. Polyclonal anti-BVDV showed Intra cytoplasmic fluorescence granules, (X100). (b) MDBK cells (negative control). (c) MDBK cells inoculated with semen samples, treated with direct fluorescence antibody technique. Polyclonal anti-BoHV-1 showed intra nuclear fluorescence granules, (X100)

Twelve (30%) positive BVDV samples out of 40 were detected by direct fluorescent antibody technique. Positive samples showed green Intracytoplasmic fluorescence granules as shown in Figure 2(a). While direct fluorescent antibody technique for detection of BoHV-1 showed intranuclear fluorescence granules in 8/40 (20%) positive samples as appeared in Figure (2c) parallel with control sample in Figure 2(b).

### 3.3. Detection of viral nucleic acids using real-time PCR assays

**Figure 3.**
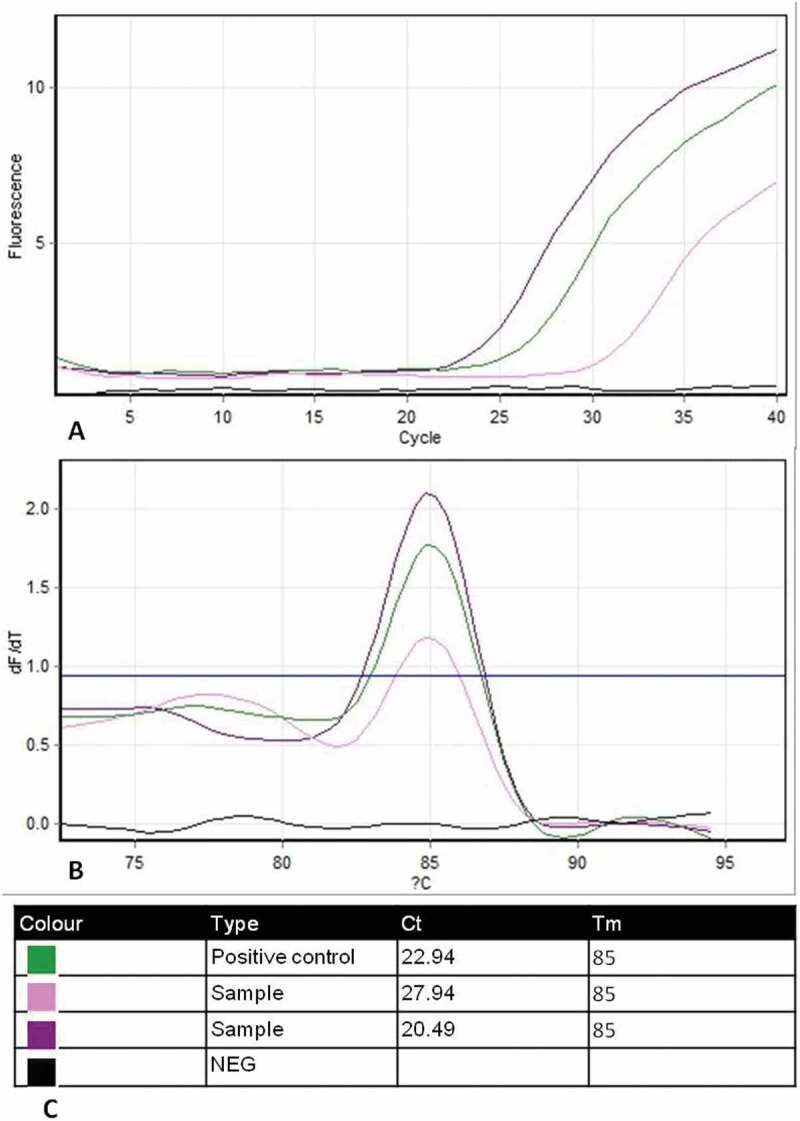
Amplification and melting (dissociation) plots of BVDV samples using SYBR green-based RT-PCR assay. (a) Amplification curves of positive samples with BVDV-NADL reference strain as positive control (cycle number is plotted against relative fluorescence units). (b) Melting curves analysis of amplified products yielded one expected dissociation peak of Tm = 85°C corresponding to reference strain (Temperature on the x-axis is plotted against the negative derivation of the measured fluorescence signal on the y-axis subsequent to amplification). (c) Outline table includes identification of curve colour, Ct and Tm for each tested sample

**In BVDV detection assay**, 14 **(35%)** samples were successfully amplified by SYBR green real-time PCR technique out of 40 tested samples. All amplified samples yielded fluorescent signals indicating positive amplification of a specific PCR product of 208 bp fragment of the 5ʹ un-translated region (5ʹ UTR) of the pestivirus genome (Figure 3a). Cycle threshold (Ct) values of positive samples ranged from 20.49 to 27.94 compared with Ct value of 22.94 for the positive control (Figure 3c). No fluorescent signals were recorded in negative control samples. Following the amplification, melting analysis of the amplified genome segment of BVD-NADL reference strain and positive samples gave one expected dissociation peak of Tm 85°C, indicating specific amplification (Figure 3b).

**Figure 4.**
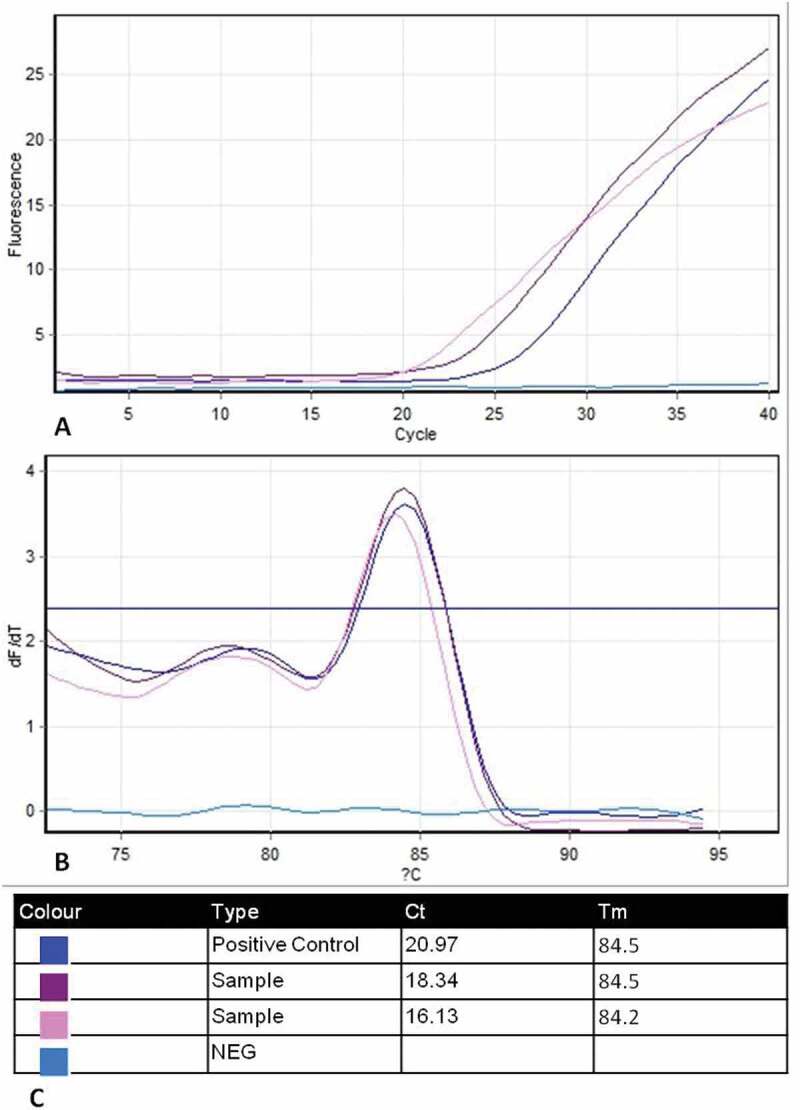
Amplification and melting plots of BoHV-1 samples using SYBR green-based PCR assay. (a) Amplification curves of positive samples with BoHV-1Colorado reference strain as positive control (cycle number is plotted against relative fluorescence units). (b) Melting curves analysis of amplified products yielded one expected dissociation peak of Tm = 84.5°C corresponding to reference strain (Temperature on the *x* axis is plotted against the negative derivation of the measured fluorescence signal on the *y* axis subsequent to amplification). (c) Outline table includes identification of curve colour, Ct and Tm for each tested sample

**BoHV-1 detection assay** revealed that 8/40 **(20%)** positive samples with Ct values from 17.35 to 31.48 and 20.97 for the positive control (Figure 4a). The specificity of positive samples was confirmed by melting curve analysis which indicated Tm 84.5°C, for a 97 base-pair sequence amplicon of the glycoprotein B (gB) gene, corresponding to a positive control (Figure 4b).

### 3.4. Comparison between different methods adopted for viral detection

Positive and negative samples as declared by different diagnostic tests are shown in Table 1.

**Table 1.** Comparison between different tests used for detection of both BVDV and BoHV-1 in semen samples

Test	Total No. of samples	BVDV		BoHV-1	
		No of positive samples	No. of negative samples	No of positive samples	No. of negative samples
Virus isolation	40	-	40	8	32
FAT		12	28	8	32
Real- time PCR		14	26	8	32

### 3.5. Effect of BVDV and/or BoHV-1 infection on fresh semen parameters

Based on the results of real-time PCR assay, samples were divided into four groups, namely: virus-free, BVDV infected, BoHV-1 infected and both viruses infected (mixed) groups. As shown in Table 2, the standard error of the mean (±SEM) of evaluated fresh semen parameters of samples in each group was recorded. Statistical analysis indicated a significant decrease in sperm motility, liveability and concentration with a significant increase in sperm abnormalities at p-value <0.01 between each of BVDV infected, BoHV-1 infected and both viruses infected group (mixed) versus the virus-free group. However, no significant difference was recorded between these three infected groups.

**Table 2.** Effect of viral infection on fresh semen quality (Mean ± SEM)

Group	No. of samples	Motility (%)	Abnormality (%)	Live/dead (L/D) (%)	Concentration (×106/ml)
BVDV	10	40.63 ± 2.20 ^a^	37.88 ± 2.22 ^a^	48.13 ± 2.82 ^a^	7.85 ± 0.23 ^a^
BoHV-1	4	38.75 ± 2.39 ^a^	39.75 ± 2.02 ^a^	41.25 ± 3.15^a^	7.93 ± 0.34 ^a^
Mixed	4	35.00 ± 6.77 ^a^	35.25 ± 2.78 ^a^	45.00 ± 7.10 ^a^	7.08 ± 0.30 ^a^
Free	26	76.04 ± 1.50 ^b^	13.17 ± 0.41 ^b^	84.17 ± 1.27 ^b^	8.34 ± 0.10 ^b^

Values with different superscripts in the same column differs significantly at P < 0.01.

## 4. Discussion

Routine monitoring of semen donors under official veterinary control has been established by governments worldwide to avoid the use of contaminated sperm in assisted reproduction and prevent the spread of diseases [30]. BVDV and BoHV-1 are two economically significant viral infectious agents which could be transmitted via bovine semen during natural mating as well as, through artificial insemination [31]. Also, Oguejiofor et al. [8] reported their implication on poor semen quality of infected bulls.

Various methods have been described for the detection of semen-transmittable viruses. Virus isolation, when highly standardized, is considered the gold standard for virus detection. But, it is necessary to ensure that the cell cultures and medium constituents provide a very sensitive means and are not influenced by low virus levels and sample quality particularly semen. Fortunately, the application of antigen detection assays like immunofluorescence and real-time RT-PCR could overcome the limitations of virus isolation and verify high diagnostic sensitivity [23]. Molecular diagnostic methods, as rapid and reliable assays, are being progressively more used for the detection of abundant viral pathogens. Applying real-time PCR assay in diagnosis limits the risk of cross-contamination as it does not require post-PCR analysis. Additionally, it is an essential diagnostic tool providing consistent and reproducible results in comparison with the conventional PCR [32,33].

In this study, semen samples were processed as described in OIE [23], to decrease the virucidal and cytotoxic properties of seminal plasma on cell culture. Considering the BVDV isolation, no cytopathic effect (CPE) was observed after three passages; however, the presence of BVDV antigen was confirmed in 12 (30%) samples out of 40 by direct FAT. This variation may be correlated with that noncytopathic (NCP) viruses are frequently isolated from field cases in comparison with cytopathic (CP) viruses [34,35]. This is in accordance with a previous study by Pogranichniy et al. [36] who reported that the NCP biotype was most commonly isolated in their study representing 75% of the positive cases.

In the molecular investigation by two steps SYBR Green I real-time RT-PCR, 48 h inoculated semen samples in cell culture were used for nucleic acid extraction to resolve the inhibitory actions of raw semen on reverse transcriptase enzymes during amplification procedure. BVDV 190-F & V326 primers identify a 208 bp segment of BVDV nucleic acid in the 5 ‘UTR. This genome area reflects a high level of conservation and is therefore acceptable for the identification of a diverse range of pestiviruses [37,38]. The assay detected 14 (35%) positive BVDV samples out of 40. The specificity of PCR products was confirmed by melting curve analysis on the amplified products which showed only a single peak in the melting peak chart with Tm 85°C corresponding to the reference strain. No primer-dimers or nonspecific products were expressed. Sultan et al. [39] obtained 10.4% positive samples for BVDV using multiplex real-time RT-PCR. Their results declared that the sensitivity of real-time is better than that of conventional RT-PCR where positive samples with Ct values >30 were negative by the conventional RT-PCR assay. They attributed this to the extremely low viral load in the majority of the positive samples which could only be detected by the very sensitive real-time RT-PCR assay. Concurrent qRT-PCR and virus isolation analysis indicated that qRT-PCR had sensitivity 100 to 1000 times higher than the detection limit for virus isolation methods [40]. Another study used the semen of persistently infected (PI) bulls failed to isolate BVDV in cell culture although the virus had been detected by PCR [41].

In BoHV-1 detection methods, clear CPE was observed in **8** (20%) samples out of 40 after three successive passages. Virus isolation was considered negative if no cytopathic effect was recorded after three consecutive passages [42]. The existence of CPE in any passage in cell culture was considered to indicate the presence of cytopathic agent and subsequently, the existence of agents was further verified by the use of direct fluorescent antibody technique. Jain et al. [43] supported our findings and detected BoHV-1 antigen in 32.67% of the semen samples from cattle and buffaloes by FAT. Testing semen by SYBR Green I real-time PCR assay for BoHV-1 also revealed 8/40 (20%) positive samples with Ct values ranged from 17.35 to 31.48. Variations in Ct values represent a mirror of virus quantity shed in bulls’ semen that differs from animal to another. On the other hand, Rana et al. [44] obtained higher sensitivity and specificity by real-time PCR than virus isolation in the detection of BoHV-1 in extended semen and they attributed the cause to the cytotoxic effect of semen on cell culture.

By comparing results obtained by different detection methods in the current study, it was found that SYBR Green real-time PCR was the most sensitive and reliable one in the detection of BVDV, particularly in low viral load. On the other hand, virus isolation, FAT and SYBR Green real-time PCR yielded the same sensitivity in the detection of BoHV-1 in semen. These findings may be attributed to the more stability of DNA viruses during transportation and storage than RNA viruses. Unfortunately, clinical samples may be subjected to degradation by the action of host enzymes. This degradation may impair the ability of the virus to infect tissue culture and subsequently could not be isolated [45]. BoHV-1 infection has been reported in cattle after insemination with contaminated semen from seronegative donor bull at the time of semen collection. Also, semen samples from this bull were tested negative by cell culture [46]. Therefore, some laboratory tests are inadequately reliable to exclude the presence of BoHV-1. Although using FAT could be a profitable test for detection of BoHV-1 antigen within a brief period contrasting virus isolation which consumed much time [47], real-time PCR is less in time-consuming with sufficient reliability and have been credited by OIE [28].

BVDV and/or BoHV-1 contaminated semen groups in the current study showed poor semen quality including a significant decrease in sperm motility, liveability and concentration with a significant increase in sperm abnormalities. Poor semen quality could be attributed to localization of BVDV in the testes of infected bulls causing a persistent testicular infection for several weeks. Consequently, the testicular function could be impaired causing abnormalities of spermatozoa [12,41,48]. BoHV-1 excretion remains controversial [49]. It is believed that semen contamination is due to virus excretion during ejaculation in seminal plasma rather than spermatozoa. This was confirmed using a PCR-based assay [50]. On the other hand, virus antigens were detected in or on the spermatozoa’s post-nuclear cap area in the semen of naturally infected bull using FAT [51]. These studies proved that BoHV-1 has the affinity to attach to the spermatozoa’s plasma membrane causing sperm abnormalities and reduce their oocyte fertilizing ability [52]. Our findings were consistent with some studies that documented abnormalities in BVDV polluted semen, including reduced semen volume, decreased sperm concentration and motility, and increased sperm abnormalities [20,53], whereas, no defects were observed in semen or sperm content of severely infected bulls or PI bulls by Kirkland et al., [13]. Poor semen quality caused by BVDV and/or BoHV-1 may decrease semen fertilizing capacity. Sperm abnormalities were correlated with the reduction in fertility rate and its frequency was suggested to be used for prediction of semen fertilizing capacity [54,55]. Noteworthy, no significant difference in semen quality was recorded among BVDV, BoHV-1 and mixed-infected groups.

## 5. Conclusion

Semen from BVDV and/or BoHV-1 infected bulls could be a possible source of infection to vulnerable cows after natural mating or artificial insemination. Infected bulls have expressed low semen quality, which may decrease semen fertilizing capacity and reduce the rates of conception and fertility in cows. Thus, semen screening for BVDV and BoHV-1 are highly recommended for routine work, and real-time PCR was confirmed to be the ideal laboratory assay for this function.
